# Gastroduodenal Artery Pseudoaneurysm Rupture Post-Billroth II Surgery: Case Report

**DOI:** 10.7759/cureus.3833

**Published:** 2019-01-07

**Authors:** Zeinab Awada, Hassan Al Moussawi, Mira Alsheikh

**Affiliations:** 1 Internal Medicine, Lebanese American University-Medical Center, Beirut, LBN; 2 Internal Medicine, Staten Island University Hospital, Staten Island, USA

**Keywords:** gastroduodenal artery, aneurysm, gastrointestinal bleed, pseudoaneurysm

## Abstract

Visceral artery aneurysms (VAAs) and visceral artery pseudoaneurysms (VAPAs) are defined as more than a 1.5 fold increase in the normal diameter of the celiac, superior, or inferior mesenteric arteries and their branches. They represent a rare finding with an incidence ranging between 0.1% to 0.2%.

Depending on the mechanism of formation, aneurysms can be divided into true aneurysms or pseudoaneurysms. True aneurysms involve all layers of the wall, which are usually thinned but remain intact and commonly result from vessel wall abnormalities. However, pseudoaneurysms occur after vascular injuries or nearby inflammatory process causing a tear in the vessel wall. Pancreatitis is the most common cause of pseudoaneurysm. Nevertheless, other conditions, such as autoimmune disorders, vascular interventions, laparoscopic cholecystectomy, and even hepatic transplantation, have been reported to increase the risk of pseudoaneurysm formation. Herein, we are reporting a case of a gastroduodenal artery pseudoaneurysm rupture in a patient with altered anatomy secondary to Billroth II surgery.

## Introduction

Pseudoaneurysms are most frequently recognized between 50 and 58 years of age [1]. They are more commonly seen involving the splenic, renal, hepatic, and the pancreaticoduodenal arteries. Only 1.5% of all reported visceral artery aneurysms (VAAs) involve the gastroduodenal artery (GDA) [1]. While only 7.5% of GDA aneurysms are asymptomatic, an acute rupture was found to be the most common clinical presentation and is recognized as a life-threatening condition [2].

## Case presentation

A 56-year-old male patient, an ex-smoker, non-alcoholic with a past medical history of hypertension, coronary artery disease, end-stage renal disease, and adrenal insufficiency, presented for fever secondary to left foot cellulitis of one week's duration. His past surgical history was significant for a Billroth II surgery one-year prior to presentation for a bleeding peptic ulcer. The patient was started on cefazolin after which he improved clinically and was planned to be discharged four days after hospitalization.

One day prior to discharge, he developed an episode of hematemesis. Gastroscopy showed a normal esophagus, normal-appearing afferent and efferent limbs, and mildly localized erythema at the level of gastrojejunal anastomosis with no evidence of blood or recent bleeding. However, the patient had several episodes of hematemesis and melena the next day which was complicated by hemorrhagic shock. After resuscitation, an urgent gastroscopy was done again which showed active bleeding in the efferent loop and a visible vessel at the level of the cardia that was clipped and injected with adrenaline.

**Figure 1:**
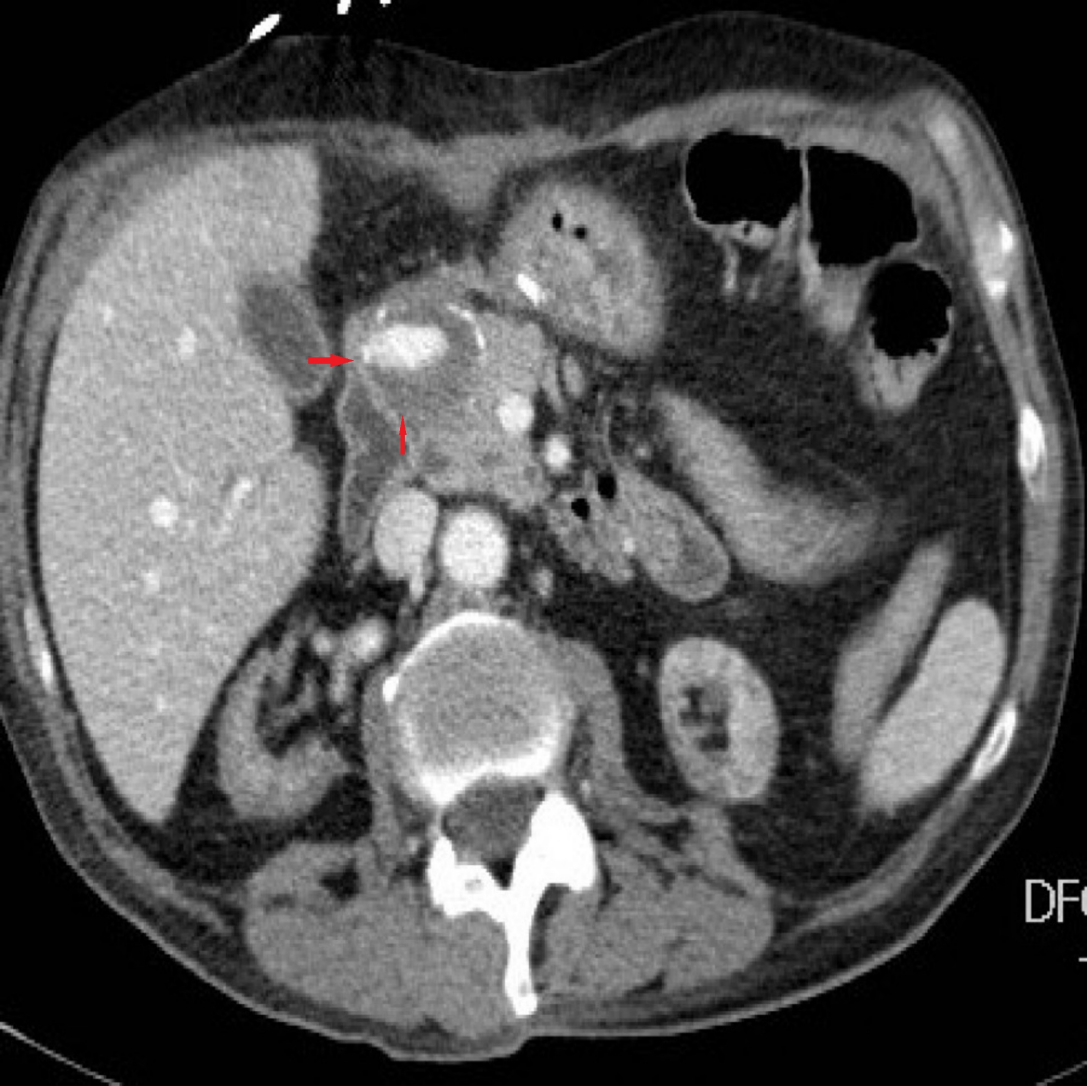
Contrast-enhanced computed tomography (axial view) showing contrast material extravasation from gastroduodenal artery pseudoaneurysm

**Figure 2:**
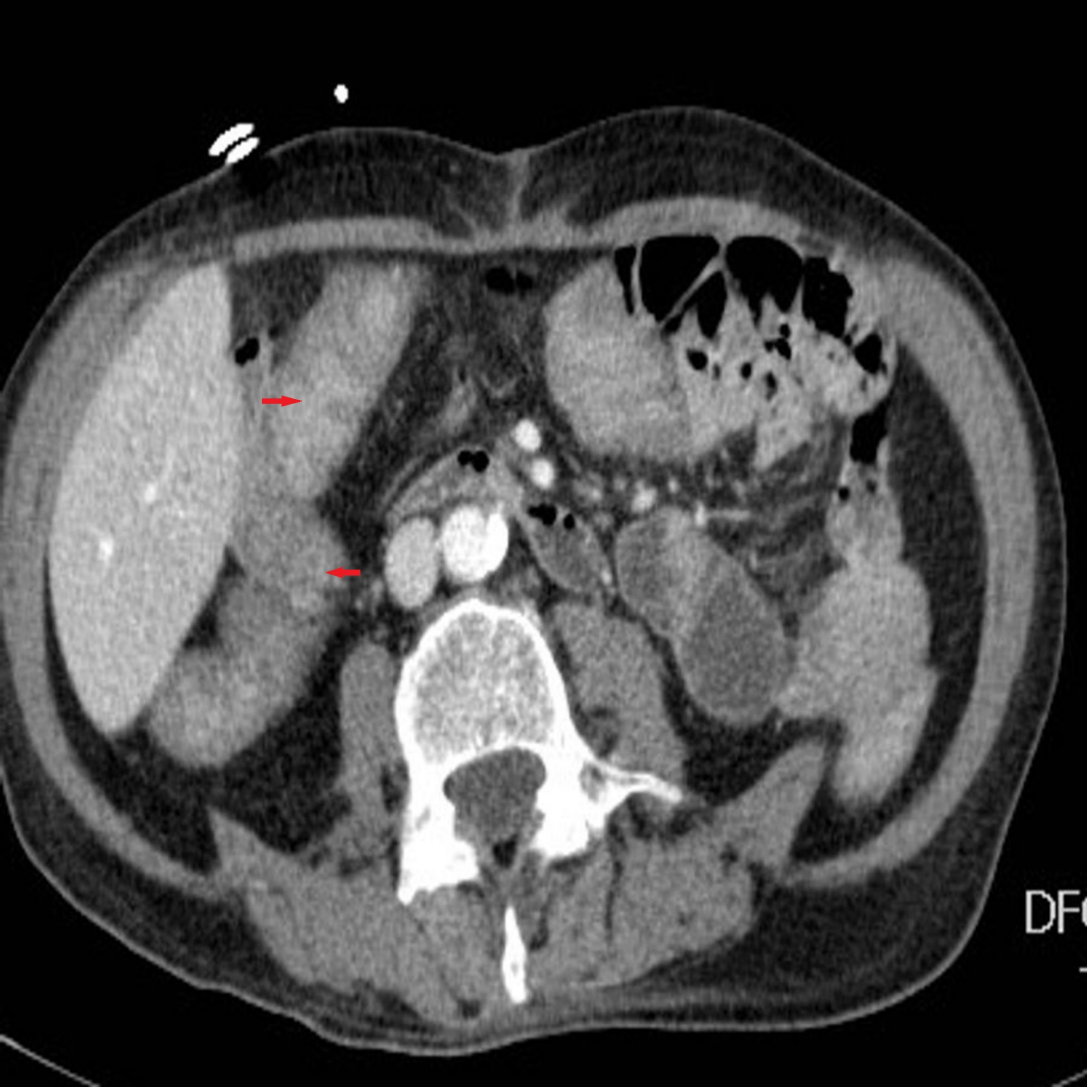
Computed tomography (CT) of the abdomen (axial view) showing blood in the small intestine

**Figure 3:**
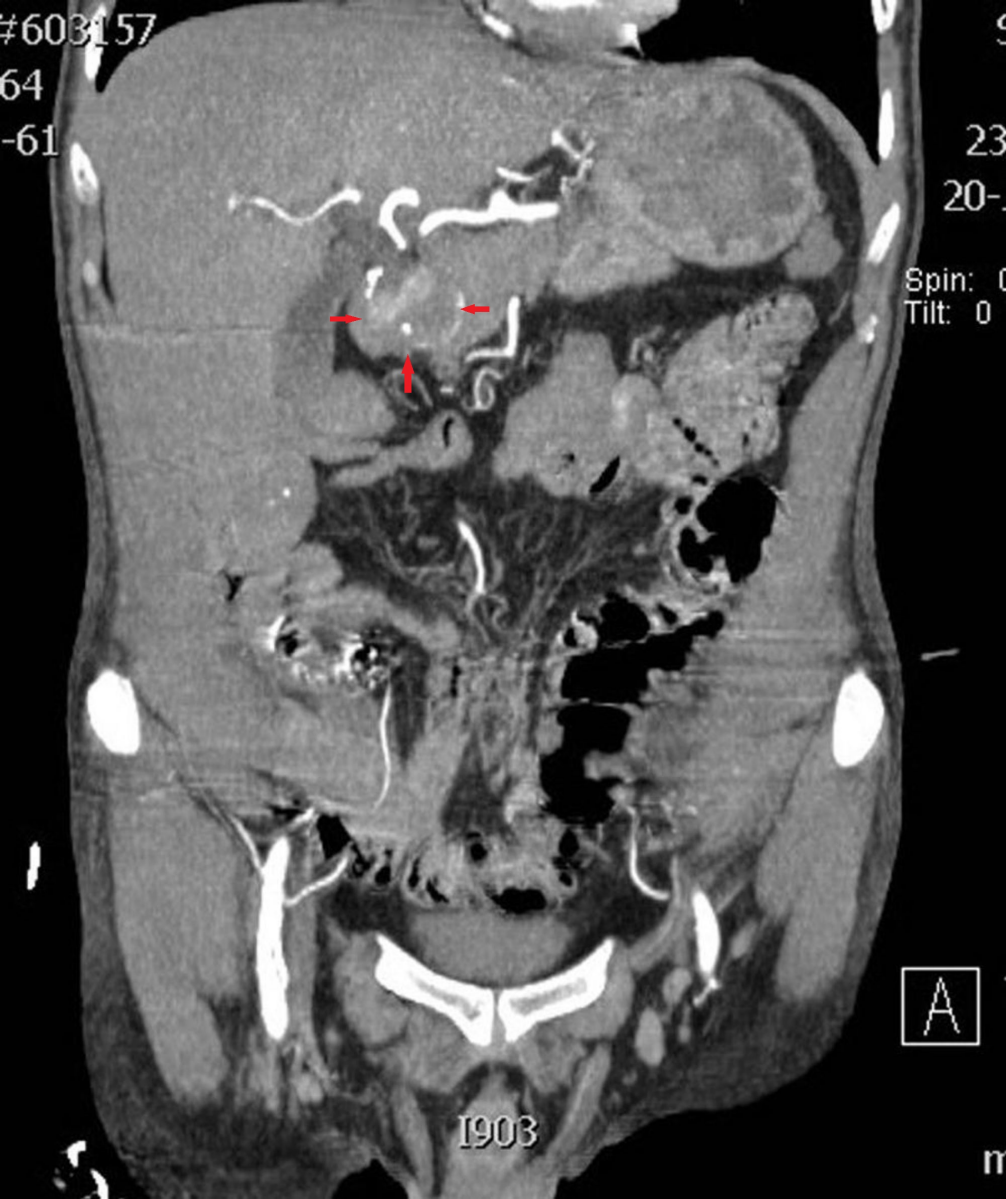
Computed tomography (CT) of the abdomen (axial view) showing blood in the small intestine

Later on, during the same day, the patient again developed massive hematemesis associated with melena. An urgent computed tomography angiography (CTA) of the abdomen/pelvis was done which showed extravasation of the contrast material near the head of the pancreas that could represent a hemorrhagic site at the efferent segment, as well as a 2.5 cm bleeding pseudoaneurysm at the gastroduodenal artery (Figures 1-3).

An urgent laparotomy was done, and a bleeding pseudoaneurysm of the gastroduodenal artery was identified. Vessel ligation was successful in controlling the source of bleeding, and the patient was discharged home a few days later.

## Discussion

A pseudoaneurysm, also known as a false aneurysm, is the result of an injury to the layers of a vascular wall by which blood leaks through the vessel wall but remains contained by a fibrous capsule since all three layers of the arterial wall are disrupted. It can be a consequence of an inflammation which results in vascular wall destruction, a scenario most frequently seen with pancreatitis. Almost 10% of patients with pancreatitis develop pseudoaneurysms of the pancreatic arteries [3]. However, blunt and penetrating abdominal trauma, iatrogenic injury from instrumentation, and mainly operative trauma during gastrectomy for duodenal ulcer can also contribute to its formation. In our case, it is likely that the pseudoaneurysm formation was related to an iatrogenic injury (mainly operative trauma during gastrectomy for his refractory peptic ulcer), which caused altered anatomy secondary to surgery.

Even though a GDA pseudoaneurysm can be asymptomatic, some patients can suffer from abdominal pain, compressive symptoms (nausea, vomiting), or a pulsatile abdominal mass/bruit with a rupture causing hematemesis, melena, or even shock, being the most serious and even fatal presentation.

Due to the poor support of the pseudoaneurysm wall and its rapid growth, the risk of rupture is higher than that of a true aneurysm of comparable size. This warrants early diagnosis and treatment due to the high mortality rates associated with rupture ranging from 25% - 70% [4]. Hence, once identified, all gastroduodenal artery pseudoaneurysms should be actively managed and treated regardless of their size or presenting symptoms.

Visceral angiography is the gold standard diagnostic test with a sensitivity reaching 100%; it can be utilized for both diagnostic or therapeutic purposes [5]. A computed tomography (CT) scan is a great modality that shows the features of pseudoaneurysm in the majority of cases with a 67% sensitivity. Ultrasound can also be helpful; it is 50% sensitive and successfully indicates the vascular nature of the mass.

The management of a gastroduodenal artery pseudoaneurysm can be achieved either by a surgical intervention (which includes vessel ligation, aneurysmal sac exclusion, and revascularization) or via endovascular intervention where the majority of the pseudoaneurysms are successfully treated. Urgent open laparotomy is the treatment of choice when there is an aneurysmal rupture or in hemodynamically unstable patients. However, endovascular management with a variety of aneurysmal isolation techniques in clinically stable patients has shown great success rates. Percutaneous endovascular management is considered a safe and successful alternative, compared to conventional surgery, with less mortality and morbidity rates and is associated with a decreased length of hospital stay in the elective setting.

## Conclusions

Gastroduodenal artery pseudoaneurysm rupture is a rare, life-threatening condition, and bleeding into the gastrointestinal tract is the most rapidly fatal complication of an arterial visceral pseudoaneurysm. This article helps to highlight the importance of recognizing and managing a pseudoaneurysm rupture in patients presenting with symptoms of a massive gastrointestinal (GI) bleed and a history of recent pancreatitis, vascular, or laparoscopic intervention.
